# Optimization of the Elasticity and Adhesion of Catechol- or Dopamine-Loaded Gelatin Gels under Oxidative Conditions

**DOI:** 10.3390/gels8040210

**Published:** 2022-03-31

**Authors:** Florence Back, Eric Mathieu, Cosette Betscha, Salima El Yakhlifi, Youri Arntz, Vincent Ball

**Affiliations:** 1 Faculté de Chirurgie Dentaire, Université de Strasbourg, 8 Rue Sainte Elisabeth, 67000 Strasbourg, France; florence.back2@etu.unistra.fr (F.B.); salima.elyak@gmail.com (S.E.Y.); youri.arntz@unistra.fr (Y.A.); 2 Unité Mixte de Recherche 1121, Institut National de la Santé et de la Recherche Médicale, 1 Rue Eugène Boeckel, CEDEX, 67084 Strasbourg, France; ericmathieu74@yahoo.fr (E.M.); cosetta.betscha@medecine.u-strasbg.fr (C.B.)

**Keywords:** gelatin adhesives, phenolic compounds, optimization

## Abstract

The synthesis of surgical adhesives is based on the need to design glues that give rise to strong and fast bonds without cytotoxic side effects. A recent trend in surgical adhesives is to use gel-forming polymers modified with catechol groups, which can undergo oxidative crosslinking reactions and are strongly adhesive to all kinds on surfaces in wet conditions. We previously showed that blending gelatin with catechol can yield strong adhesion when the catechol is oxidized by a strong oxidant. Our previous work was limited to the study of the variation in the sodium periodate concentration. In this article, for an in-depth approach to the interactions between the components of the gels, the influence of the gelatin, the sodium periodate and dopamine/(pyro)catechol concentration on the storage (G′) and loss (G″) moduli of the gels, as well as their adhesion on steel, have been studied by shear rheometry. The hydrogels were characterized by infrared and UV-Vis spectroscopy and the size of their pores visualized by digital microscopy and SEM after freeze drying but without further additives. In terms of adhesion between two stainless steel plates, the optimum was obtained for a concentration of 10% *w*/*v* in gelatin, 10 mM in sodium periodate, and 20 mM in phenolic compounds. Below these values, it is likely that crosslinking has not been maximized and that the oxidizing environment is weakening the gelatin. Above these values, the loss in adhesiveness may result from the disruption of the alpha helixes due to the large number of phenolic compounds as well as the maintenance of an oxidizing environment. Overall, this investigation shows the possibility to design strongly adhesive hydrogels to metal surfaces by blending gelatin with polyphenols in oxidative conditions.

## 1. Introduction

**Scheme 1:**
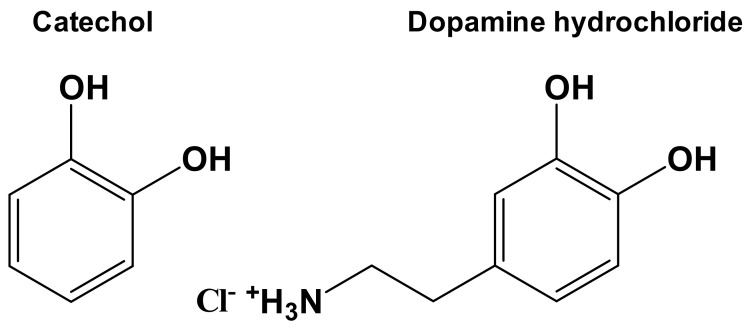
Chemical structures of catechol and dopamine hydrochloride, the two molecules used as adhesion promoters in gelatin hydrogels.

The use of hydrogels is of major importance in many technological fields such as in food science, in cosmetics, and in biomaterials science, where they can be used as templates for tissue engineering [1], drug delivery systems [2,3], and new materials for surgical adhesives [4,5,6]. In this last application field, adhesive hydrogels may offer many advantages with respect to sutures and stapples owing to their biodegradability. Nevertheless, biomolecules extracted from plants or animals present important drawbacks related to their potential immunogenicity. When fibrin gels from the patient itself are used, such concerns are avoided but at the cost of weak adhesiveness [7]. On the other hand, synthetic polymers such as cyanoacrylates offer much stronger adhesion than fibrin and are degradable; however, their degradation products, mainly aldehydes, display some cytotoxicity [8]. In the last two decades, the field of bioadhesives has undergone a revolution inspired after the adhesion mechanism of marine organisms such as mussels [9,10,11] or barnacles was understood. In all these animals, proteins containing an unusually high molar fraction of a modified L-tyrosine residue, namely L-DOPA, is present. These proteins have been found responsible for strong and irreversible (as long as the proteins are not hydrolyzed) adhesion in wet conditions and on a large repertoire of materials: metals, oxides, and polymers [12]. On titanium dioxide, the adhesion strength measured by atomic force microscopy has been found to be pH dependent, reaching values close to those expected for the rupture of covalent bonds at low pH values [13]. The same pH-dependent trend has been found for mussel foot proteins (mfp) on mica surfaces [14]. Since then, huge research efforts have been performed to understand—based on the versatile chemistry of catechols and catecholamines [15,16]—and to mimic such a strong adhesion. In almost all investigations, either catechol-containing proteins or peptides [17,18], or catechols grafted on polymer chains or incorporated in the polymer backbone [19,20,21,22,23,24,25,26,27,28,29,30,31,32], have been used. A recent review summarizes all the synthesis and adhesive properties of those catechol functionalized hydrogels [33]. However, using a different strategy, Lee and coworkers showed that the single blending of tannic acid (TA), a polyphenol widely available from leaf extracts of many plants and which has antibacterial–antioxidant properties [34], with a 4-arm poly(ethylene glycols) (PEG) afforded an adhesive strength as high as (0.17 ± 0.01) MPa on porcine skin [35]; however, it was derived from extremely concentrated solutions in PEG and TA (1.0 g·mL^−1^). In this investigation, TA was used as a hydrogen bond donor, whereas the 4-arm modified poly(ethylene glycols) (PEG) worked as a hydrogen bond acceptor to produce a strong glue, enabling a 2.5 fold enhancement of the adhesion strength between the epidermic side porcine skin with respect to fibrin glue [35]. The same effect was obtained using poly(vinyl alcohol) [36] or poly(N-vinylpyrrolidone) [37] in place of the 4-arm modified PEGs. These investigations showed the possibility to immobilize the catechol or the polyphenols as a glue in a polymer matrix by single blending and a rationale choice of the polymer in order to establish interactions with the adhesive active moiety, namely the catechol or the polyphenol. This could allow to alleviate the major drawbacks of the adhesives containing covalently bound catechols, specifically in quite sophisticated organic chemistry, which sometimes requires protection-deprotection steps in the catechol, and subsequent purification steps. Inspired by this idea of blending polymers that can undergo gelation under an external trigger with catechols, we showed the possibility to produce strongly adhesive hydrogels on steel surfaces by blending bovine gelatin with pyrocatechol. In this study, the adhesion was triggered by catechol oxidation, with sodium periodate during the gelation process itself [38]. In the previous study, gelatin B at 10% (*w*/*v*) was blended with pyrocatechol or pyrogallol (but a with weak contribution to adhesion) at 10 mM, with increasing concentrations in sodium periodate. In the case of catechol (we shall henceforth use the name catechol instead of pyrocatechol), we demonstrated a 2.7-fold increase in adhesive strength and a 21-fold increase in debonding energy for 10% *w*/*v* gelatin + 10 mM catechol oxidized with 30 mM NaIO_4_ (adhesive strength 82 ± 7 kPa, and debonding energy of 105 ± 21 J/m^2^ between two steel plates) [38]. The present study aims to focus on the variable concentration of catechol–dopamine after determining the optimal amount of oxidant and gelatin. The intrinsic mechanical properties (gelation kinetics, viscoelastic properties, and adhesion) of the different gelatin + catechol–dopamine + NaIO_4_ gels were evaluated using a rheometer. The obtained results were discussed on the basis on the possible reactions of oxidized catechol or dopamine with gelatin chains and on a morphological basis after quantitative evaluation of the pore size of the gels containing variable amounts of catechol or dopamine. For the sake of simplicity, we will adopt the following nomenclature for the investigated hydrogels: GxCyNz or GxDyNz, where G, N, C, and D represent gelatin, sodium periodate (NaIO_4_), catechol, and dopamine, respectively. In this nomenclature, x, y, and z are the weight fraction of gelatin (% *w*/*v*), the molar concentration of catechol or dopamine, and the molar concentration of NaIO_4_, respectively. The rationale between comparing catechol with dopamine was the presence of an additional ethylamine group on dopamine (Scheme 1), which may compete in an efficient manner with the nucleophilic amino acid side chains on the gelatin to allow for crosslinking.

## 2. Results and Discussion

### 2.1. Optimal Concentration Gelatin and in NaIO_4_

The gelation kinetics of gelatin were first investigated as a function of the gelatin concentration in the presence of 10 mM NaIO_4_. The gelation kinetics are displayed in Figure S1 of the Supplementary Materials. It appears that the final storage and loss moduli after 3 h of gelation display a different regime below and above 10% *w*/*v* in gelatin. Below 10% *w*/*v*, G′ scales as C^4.6±0.3^ (Figure S2 in the Supplementary Materials), whereas the influence of the concentration is less pronounced at a higher gelatin concentration, where G′ scales as C^1.8±0.2^. This power law dependence of G′ with the gelatin concentration is in agreement with other investigations performed on gelatin gels [39,40]. It may well be that steric hindrance due to too high a concentration can lead to a loss of opportunities for molecular alignment and interactions between the chains, hence causing a transition from a C^4.6^ to a C^1.8^ dependence. From the data in Figure S2 in the Supplementary Materials, it seems legitimate to assume that a concentration above 10% *w*/*v* decreases the possibility of optimal molecular alignment and formation of junctions between gelatin chains. As an interesting observation, the storage and the loss moduli scale with the same power law in the low (below 10% *w*/*v*) and in the high (above 10% *w*/*v*) concentration range. This is a strong indication of the fractal nature of the gelatin gels investigated in the presence of 10 mM NaIO_4_ [41]. Hence, a constant gelatin concentration of 10% *w*/*v* was used in all the forthcoming experiments.

**Figure 1:**
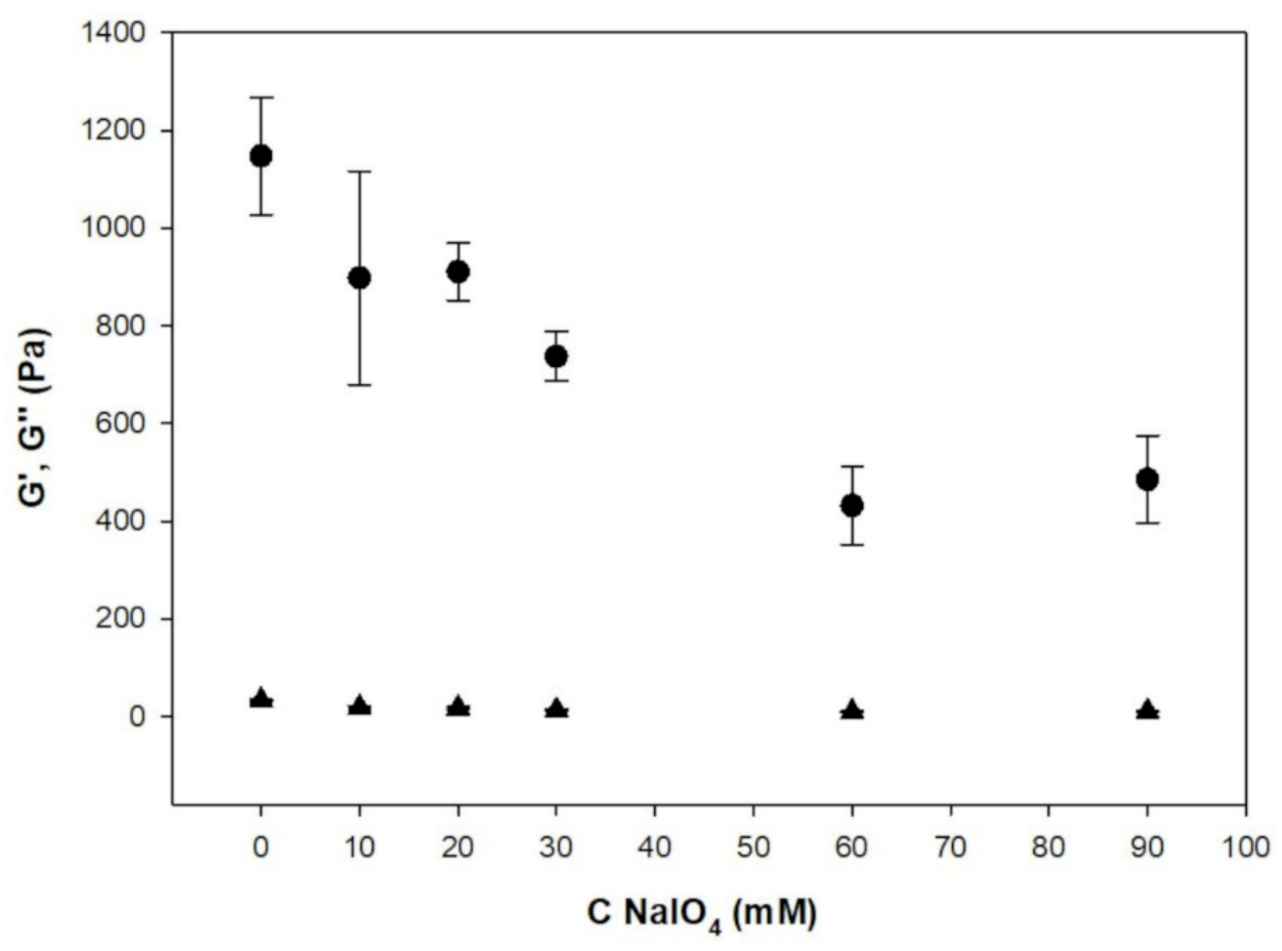
Final storage (circles) and loss moduli (triangles) of gelatin gels after 3 h of gelation as a function of the NaIO_4_ concentration added to a gelatin sol at 10% *w*/*v*. The data correspond to the average over three independent measurements and the error bars to ± one standard deviation.

We then increased the NaIO_4_ concentration, the oxidant that will be used to oxidize the introduced catechol or dopamine, in order to investigate its effect on the rheological properties of gelatin itself. Indeed, it is well known that such a strong oxidant (the redox standard potential of the IO_4_^−/^IO_3_^−^ redox couple is equal to +1.55 V vs. the normal hydrogen electrode) is able to oxidize vicinal diols or amino alcohols (N-terminal residues of serine and threonine or hydroxylysine) to produce formaldehyde [42], and such chemical modifications may modify the mechanical properties of gelatin. It is found that the storage modulus of gelatin gels, measured after 3 h of gelation, decreases progressively upon an increase in the sodium periodate concentration up to 90 mM, to reach only about 40% of the storage modulus of the pristine gelatin gel (Figure 1). Between 10 and 20 mM in NaIO_4_, the gel loses only about 20% of its elasticity compared to the pristine gelatin gel. This moderate decrease in storage modulus is acceptable from a mechanical point of view. For this reason, we will restrict our investigation to 10% (*w*/*v*) gelatin gels and a NaIO_4_ concentration of 10 mM, and the investigated variable isthe catechol or dopamine concentration.

### 2.2. Influence of the Catechol and Dopamine Concentration on the Gels’ Elasticity and Adhesiveness

**Figure 2:**
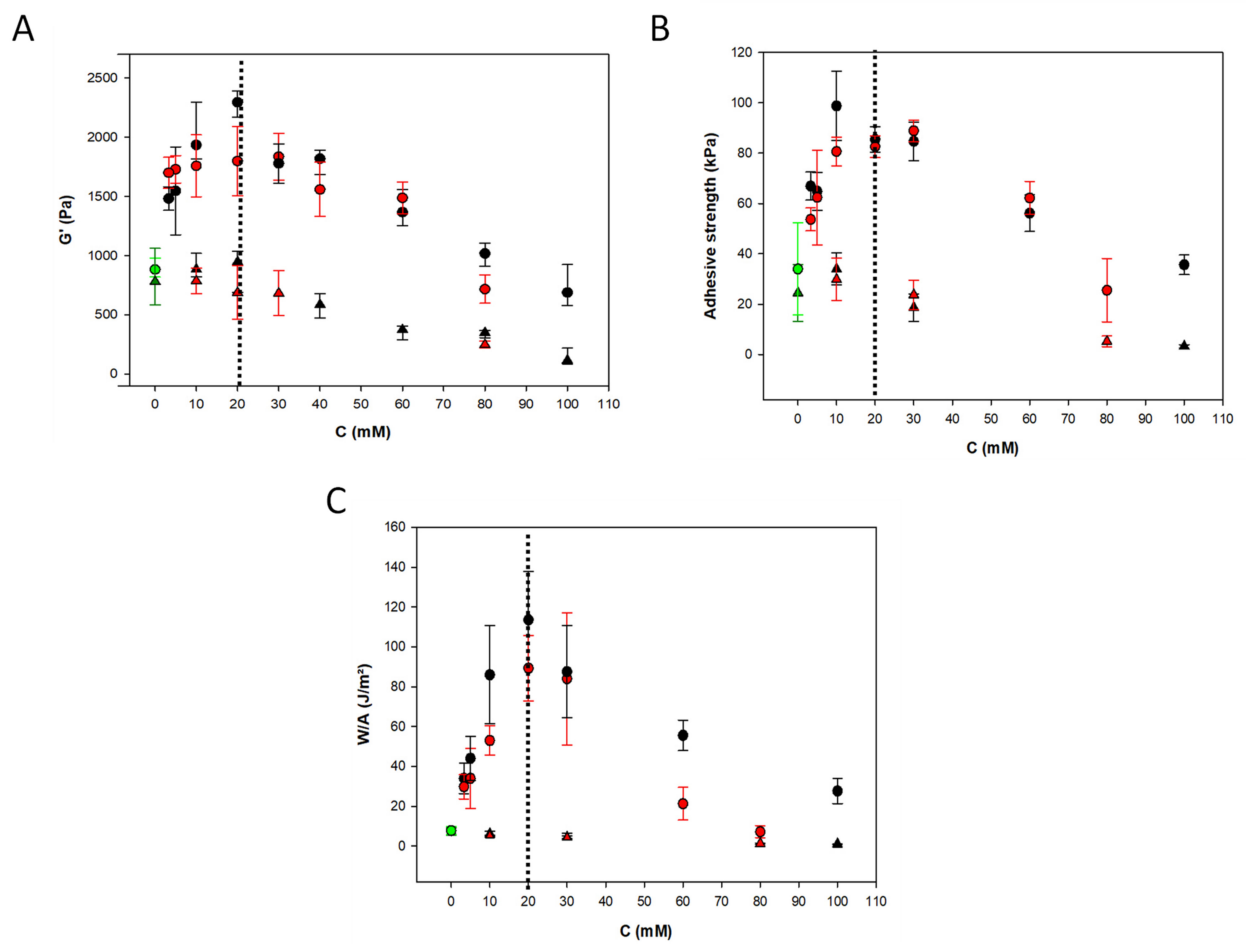
(**A**) Final storage modulus (G′) after 3 h of gelation kinetics for *G*_10_*C_y_N*_10_ (black) or *G*_10_*D_y_N*_10_ (red) as function of their concentration (circles) and the same experiments without NaIO_4_-triggered oxidation (triangles). The green circles correspond to a pure gelatin gel without NaIO_4_ and without catechol or dopamine. (**B**) Adhesion strength (**C**) and debonding energy as function of the catechol (black) or dopamine concentration (red) for gelatin 10% *w*/*v* with 10 mM NaIO_4_ (circles) or without NaIO_4_ (triangles). The green dark triangles correspond to pure gelatin gel at 10% (*w*/*v*) and the green circles to the 10% (*w*/*v*) gelatin gel but with 10 mM added NaIO_4_. In all the graphs, the error bars correspond to ± one standard deviation over three independently prepared gels. The dotted lines in all panels are aimed to qualitatively identify the optimal catechol or dopamine concentration in terms of gel elasticity and adhesiveness.

**Figure 3:**
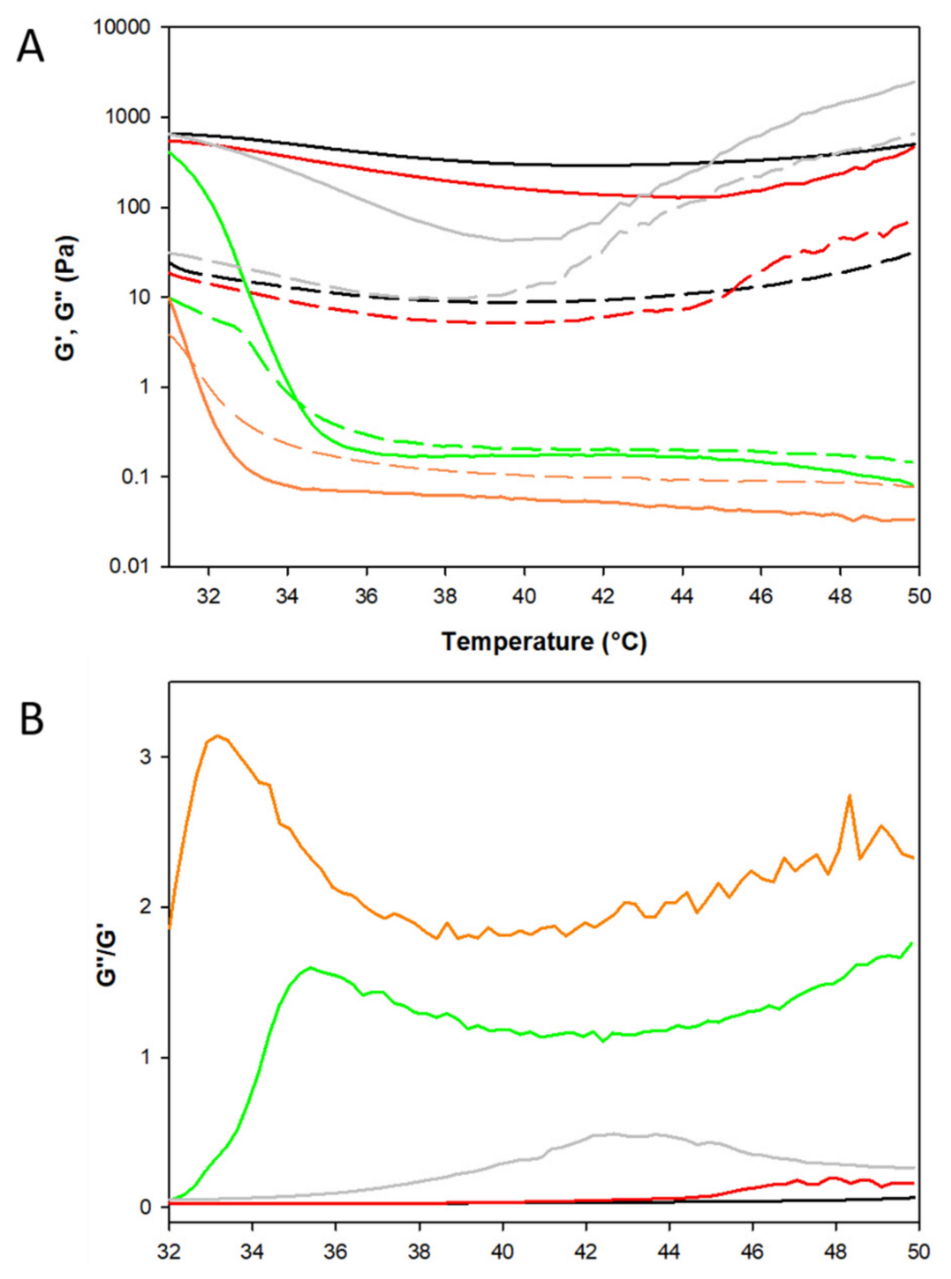
(**A**) Storage and loss moduli (G′, full lines, G″, dashed lines) of *G*_10_*N*_10_ (green), *G*_10_*C*_20_*N*_10_ (black), *G*_10_*D*_20_*N*_10_ (red), *G*_10_*C*_80_*N_1_*_0_ (gray), and *G*_10_*D*_80_*N*_10_ (orange). (**B**) Loss factor (G″/G′) of *G*_10_*N*_10_ (green), *G*_10_*C*_20_*N*_10_ (black), *G*_10_*D*_20_*N*_10_ (red), *G*_10_*C*_80_*N*_10_ (gray), and *G*_10_*D*_80_*N*_10_ (orange). The temperature sweep experiments were performed at 1 Hz and with 1% strain from an initial temperature of 25 °C to a final temperature of 50 °C at a heating rate of 1.0 °C·min^−1^.

It appears that the storage modulus of the *G*_10_*C_Y_N*_10_ and of the *G*_10_*D_Y_N*_10_ gels displays a maximum close to y = 20 mM in either catechol or dopamine (Figure 2A). The value of this maximal storage modulus amounts to 2300 ± 120 Pa and to about 1800 ± 200 Pa for catechol- and dopamine-containing hydrogels, respectively. It also appears that in the absence of NaIO_4_, specifically for the *G*_10_*C_Y_* and the *G*_10_*D_Y_* hydrogels, no maximum in the storage modulus is found but G′ decreases slowly upon an increase in the catechol or dopamine concentration. Therefore, in all cases, the addition of the oxidant increases the storage modulus (Figure 3A). It has also to be noted that the maximal storage modulus for these *G*_10_*C*_20_*N*_10_ or *G*_10_*D*_20_*N*_10_ gels is much higher than the storage modulus of the *G*_10_*N*_10_ gels, which was 1150 ± 100 Pa. The maximum in the storage modulus value for the gels where catechol or dopamine is oxidized corresponds to an oxidant/catechol or an oxidant/dopamine molar ratio close to two.

Quite interestingly, the peeling curves (some representative examples are given in Figure S3 of the Supplementary Materials) of the gels also displayed a maximal adhesion strength (Figure 2B) and a maximal debonding energy (Figure S3) for the same catechol or dopamine concentration, which was around 20 mM. In all cases of catechol- or dopamine-modified hydrogels and in the presence of 10 mM NaIO_4_, the rupture was of a cohesive nature (Figure S4 of the Supplementary Materials), meaning that the adhesion on stainless steel is extremely high and not the limiting factor of the adhesive behavior of the developed glue. Our findings highlight the fact that the most elastic gels are also the most adhesives ones, which is an optimal situation [12].

Interestingly, the catechol-containing hydrogels display an adhesive quality which is slightly larger (by about 20% at the optimal NaIO_4_ concentration of 10 mM) than the dopamine-containing ones. This trend is conserved over the whole investigated concentration range in either catechol or dopamine (Figure 2C). This point will be discussed later, based on the oxidation mechanisms of catechol and dopamine.

The values of the optimal adhesion strength obtained in this investigation, close to 100 kPa, must be compared with data from the literature. Most of these data, with the exception of the data of TA blended with 4-arm PEGs [35], poly(vinyl alcohol) [36], or poly(vinylpyrollidone) [37], or data from our previous investigation [38], were obtained for polymers with incorporated catechols or catechols grafted as side chains. It appears from Table 1 that the adhesives developed by blending a polymer able to undergo gelation with a polyphenol such as TA or with catechol are *competitive with those where the catechol unit is incorporated in the polymer chain or grafted as a side chain on the polymer backbone*. To our knowledge, only poly((3,4-dihydroxymandelic acid)_x_-co-(lactic acid)_1−x_) used to glue aluminium or steel plates displays adhesion strengths higher by one order of magnitude [29]. Our easy, one-pot preparation method may hence allow to bypass the complicated modification of polymers or the incorporation of catechol groups in the polymer backbone.

This interesting finding could be the starting point of the design of polymers able to undergo gelation and to interact with catechol or other polyphenols such as condensed polyphenols. More investigations are of course required to confirm this trend.

**Scheme 2:**
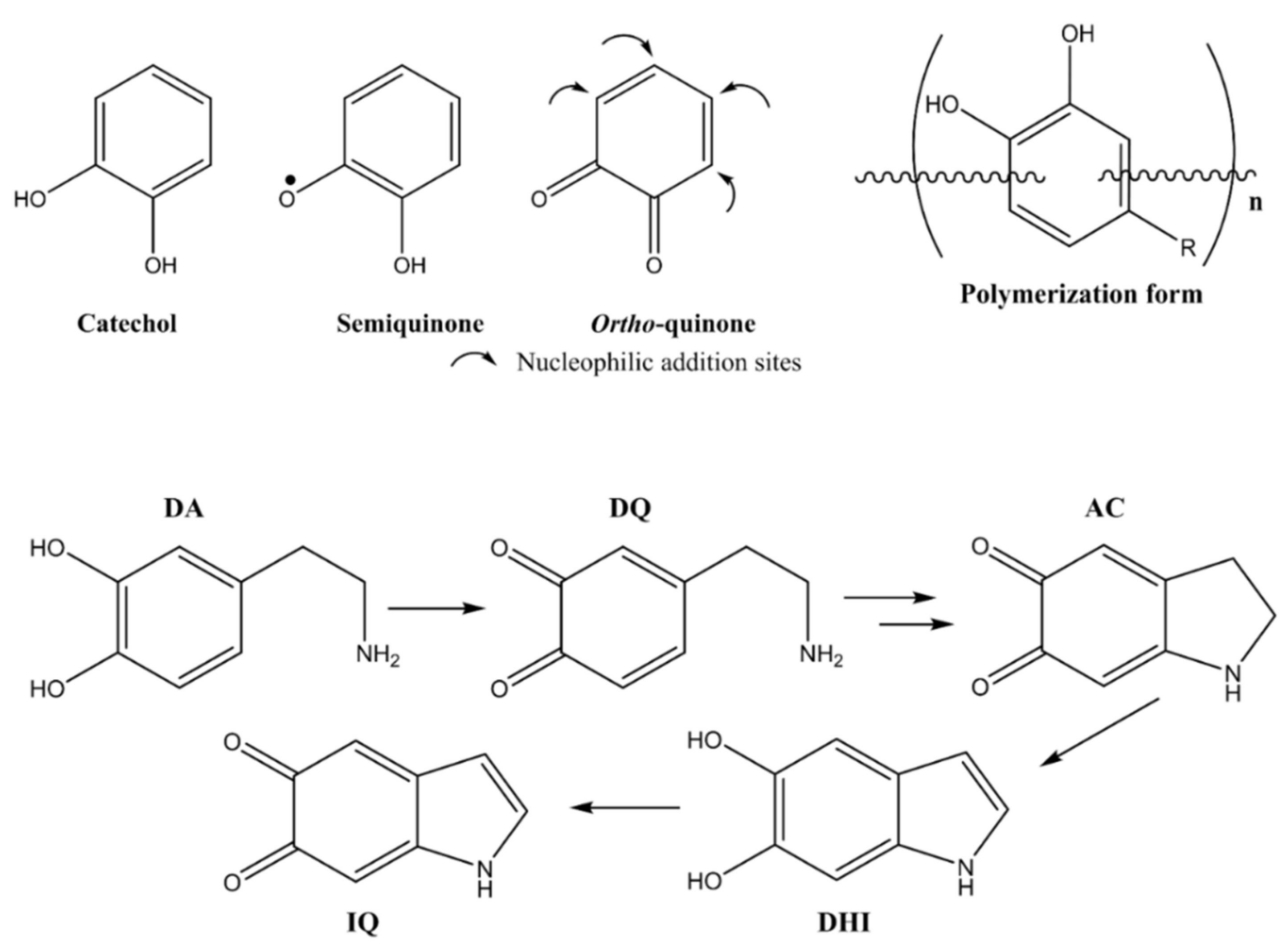
Oxidation pathways of catechol and dopamine (DA) and identification of the formed species [6,43].

We will now explore explanations as to why an optimal catechol or dopamine over NaIO_4_ ratio allows for a simultaneous maximum in the gels elasticity and their adhesiveness, whereas a gel containing higher amounts of catechol and dopamine only slightly outperforms a pristine gelatin gel (Figure 2). To begin to find such an explanation, we performed some temperature sweep experiments on the *G*_10_*N*_10_, *G*_10_*C*_20_*N*_10_, and *G*_10_*C*_80_*N*_10_*,* and the same compositions in the case of dopamine (Figure 3). These gel compositions correspond to those before the occurrence of the optimal properties, at the optimal conditions, and in the worst conditions, where a lot of catechol or dopamine is consumed with a deterioration of the mechanical properties. It appears that the *G*_10_*C*_20_*N*_10_ and *G*_10_*D*_20_*N*_10_ hydrogels remain in the gel state even when heated to 50 °C, which is interesting for biological applications because this stability range exceeds the human body temperature. As is well known from the literature [40], gelatin gels from mammals without added catechol or dopamine undergo a gel–sol transition at around 30–34 °C depending on the gelatin type (gelatin *A* or *B*), the pH, the molecular mass of the gelatin, and other physicochemical conditions. Interestingly, the *G*_10_*C*_80_*N*_10_ hydrogels remain stable up to the highest investigated temperature whereas the *G*_10_*D*_80_*N*_10_ gels undergo the gel–sol transition at a temperature which lies even below the gel–sol transition temperature of *G*_10_*N*_10_ hydrogels. This highlights, as already suspected from the data in Figure 2C, that higher concentrations in catechol are more beneficial than higher dopamine concentrations in improving the thermomechanical properties of the gelatin-based hydrogels. Even if the hydrogels behave similarly at low added catechol or dopamine levels (Figure 2), a marked difference is found for the dopamine-rich gels concerning the debonding energy (Figure 2C) and the thermal stability (Figure 3). At this point, we can make two assumptions:

(1) The mechanical and adhesive properties of the catechol- or dopamine-containing *G*_10_*C_z_N*_10_ or *G*_10_*D_z_N*_10_ hydrogels are optimal for a molar concentration corresponding to a 2-fold molar excess of catechol or dopamine with respect to NaIO_4_, and correspond to an optimal crosslinking density with gelatin. A priori, such a ratio is unexpected because the oxidation of catechol or dopamine produces a quinone with the loss of two protons and two electrons, whereas the IO_4_^−^ anion is reduced in the IO_3_^−^ anion in a two electron process. One would hence expect an optimal catechol or dopamine concentration close to 10 mM, and hence a catechol/dopamine ratio equal to 1. The finding that it occurs close to 20 mM might be due to the fact that the oxidation of catechol or dopamine by NaIO_4_ is a fast reaction (as observed in pure solvent, see also [43]), and its rate limiting step is the encounter between catechol or dopamine and the oxidant in a confined and viscous medium as a gelatin-based sol. In the case of higher catechol or dopamine concentrations, those molecules do not have enough oxidant available to undergo oxidation and react preferentially with the quinones or semiquinones formed upon oxidation to yield catechol-containing aggregates or polymers rather than catechol or dopamine molecules binding on nucleophilic sites present on the gelatin. This excess of introduced catechol or dopamine is hence useless to further crosslink the gels. For catechol or dopamine concentrations lower than 20 mM, there are not enough quinones able to react with nucleophiles on gelatin, and there remains the possibility to increase the thermomechanical properties of the hydrogel by adding more solutes able to undergo oxidation.
(2) The difference between catechol and dopamine at high concentrations (above the optimal value of 20 mM) (Figure 2C) is probably related to the difference in reactivity of both molecules with nucleophiles in oxidizing conditions (Scheme 2).

**Figure 4:**
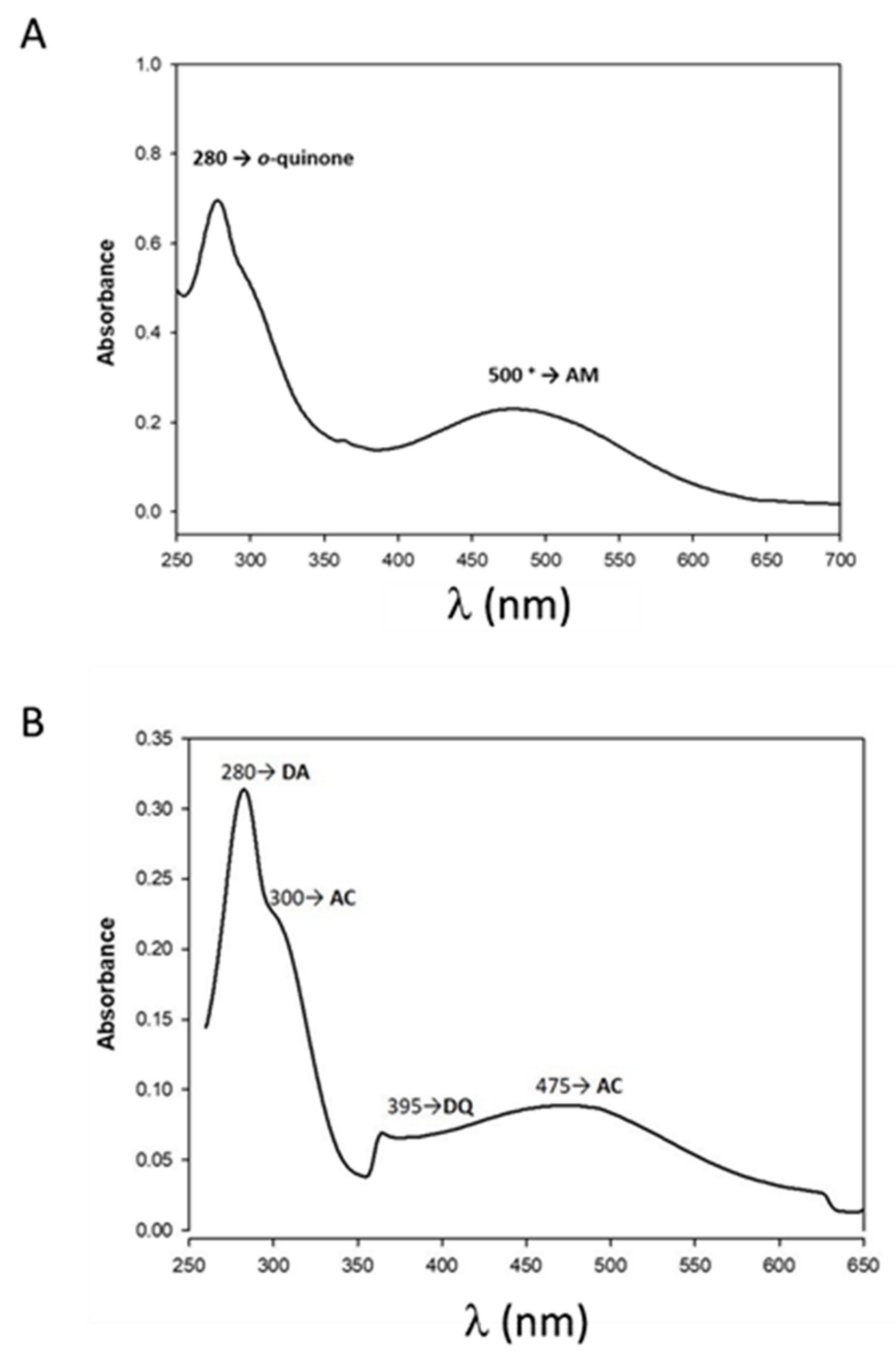
(**A**) UV-visible spectrum of *G*_10_*C*_10_*N*_10_ sol. (**B**) UV-visible spectrum of *G*_10_*D*_20_*N*_10_ sol. The samples were taken directly from the batch at elevated temperature, hence in the sol state. The nomenclature of the chemical species is the same as in Scheme 2 and AM is the abbreviation for Michael addition.

To confirm this expectation, we measured the UV-Vis spectra of hot sol solutions after mixture of gelatin with 10 mM NaIO_4_ and the addition of either catechol or dopamine (Figure 4). Significant differences were observed between catechol- and dopamine-containing samples. The spectra of the dopamine-containing mixtures display the presence of dopamine quinone (DQ) and aminochrome (AC) whereas the spectrum of the catechol-containing samples shows (a few seconds after mixing of the components) a peak at around 500 nm which can reasonably be attributed to Michael addition reactions [44] with gelatin. Such a peak is also present in the spectrum of the *G*_10_*D*_20_*N*_10_ sol (Figure 4). Note that the spectrum of the *G*_10_*D*_20_*N*_10_ sol does not display the presence of 5, 6-dihydroxyindole (DHI in Scheme 2) or 5, 6-indolequinone (IQ in Scheme 2). This is unsurprising; indeed, it has been demonstrated that the formation of DHI is delayed in the oxidation pathway of dopamine [43].

**Figure 5:**
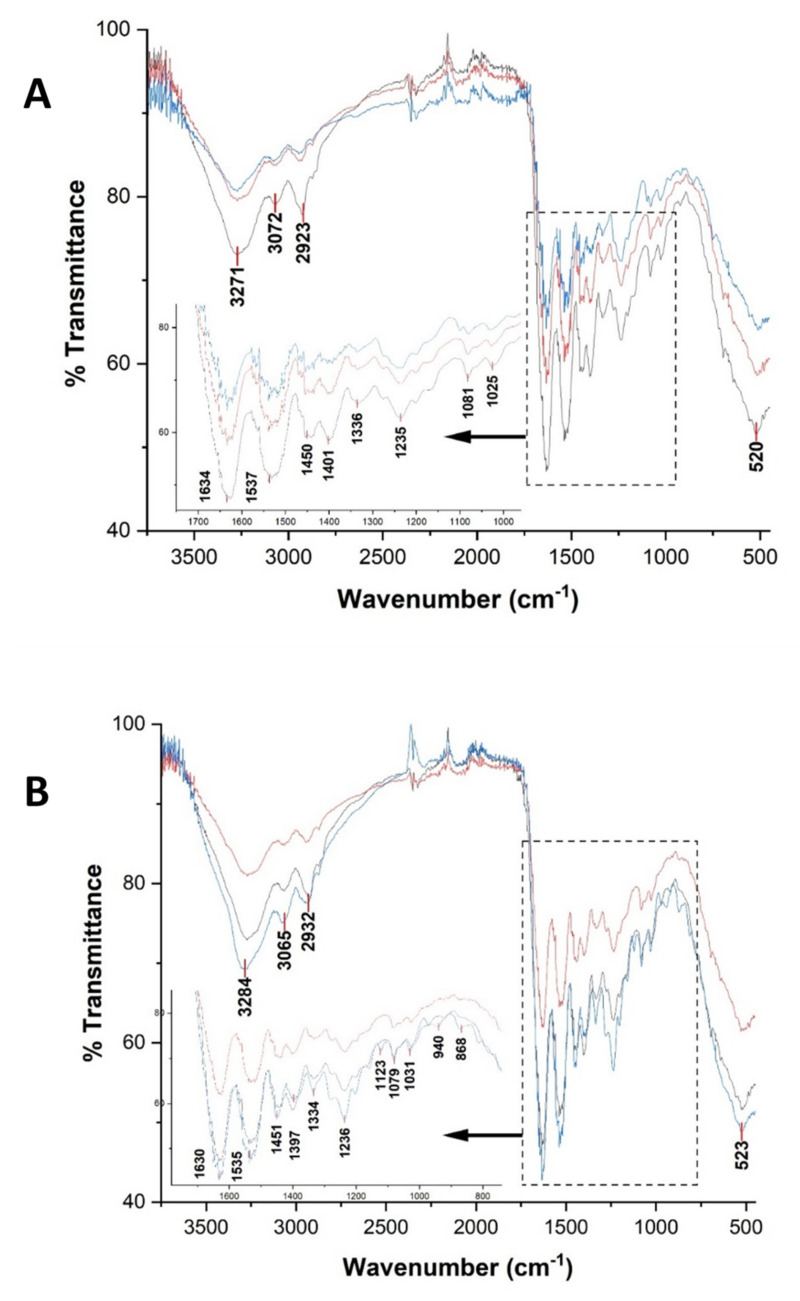
IR spectra of (**A**) *G*_10_*N*_10_, *G*_10_*C*_20_*N*_10_, and *G*_10_*C*_80_*N*_10_, and of (**B**) *G*_10_*N*_10_, *G*_10_*D*_20_*N*_10_, and *G*_10_*D*_80_*N*_10_. When the concentration of phenolic compound is high (80 mM), a difference is observed between the spectra, which corresponds only to the presence of the phenolic compounds.

To gain additional information about the formation of Michael addition or Schiff base formation between the oxidized catechol or dopamine species, we also performed some characterization by means of infra-red spectroscopy. Some typical spectra are displayed in Figure 5. The small amount of sites giving rise to covalent bonds makes it impossible to perceive any difference between the spectra of non-crosslinked gelatin (*G*_10_, *G*_10_*N*_10_, and *G*_10_*D*_80_*N*_10_) and the others [45]. However, the increased mechanical, adhesive, and thermal properties already provide evidence of crosslinking. It is customary to use the ratio of peak intensities at 1235 nm (amide III) and 1450 nm (proline and hydroxyproline) to determine the degree of alpha chains perturbation in gelatins or collagens [46]. In the case of collagen, the ratio of these peaks is equal to one, when the chains are in their native state. The analogous application for bovine gelatin type B gives a ratio (I1450 cm^−1^/I1235 cm^−1^) of 0.9701. The latter was the basis for evaluating the percentage of disruption given by the addition of phenolic compounds and periodate (Table S1 of the Supporting Information). It was found that the addition of catechol or dopamine (3.3 mM) with three equivalents of periodate did not significantly change the peak ratio (0.02% and 0.35% difference for catechol and dopamine, respectively). The addition of periodate alone (10 mM) results in a change of 2.2%, which corroborates Figure 1, where only a modest decrease in storage modulus was found in these conditions. The other concentrations of phenolic components with 10 mM periodate induce an increase in I1450 cm^−1^/I1235 cm^−1^) ratio from 1.2% to 2.8% for all species combined except for the *G*_10_*D*_80_*N_1_*_0_ hydrogel, which shows a notable variation of 4.6%. It then becomes possible to assume that the addition of phenolic species somewhat disrupts the α chains of gelatin. The excess of dopamine above the optimal concentration of 20 mM may also be implied in the formation of polydopamine micro or nanoparticles, which may not contribute to the gels’ stabilization or to the crosslinking process with gelatin [47].

To infer such a possibility and more generally to produce the influence of the added catechol or dopamine on the morphology of hydrogels, we performed a morphological characterization, combining optical reflection microscopy and scanning electron microscopy (SEM). Indeed, a change in the pore size and in the pore volume of porous materials has a strong influence on their mechanical properties [48]. One would expect that the larger the pore size, the weaker the elasticity and the gel cohesion will be; however, this did not occur in the present case, as will be shown in the following.

### 2.3. Morphological Characterizations of the Catechol- and Dopamine-Containing Hydrogels

**Figure 6:**
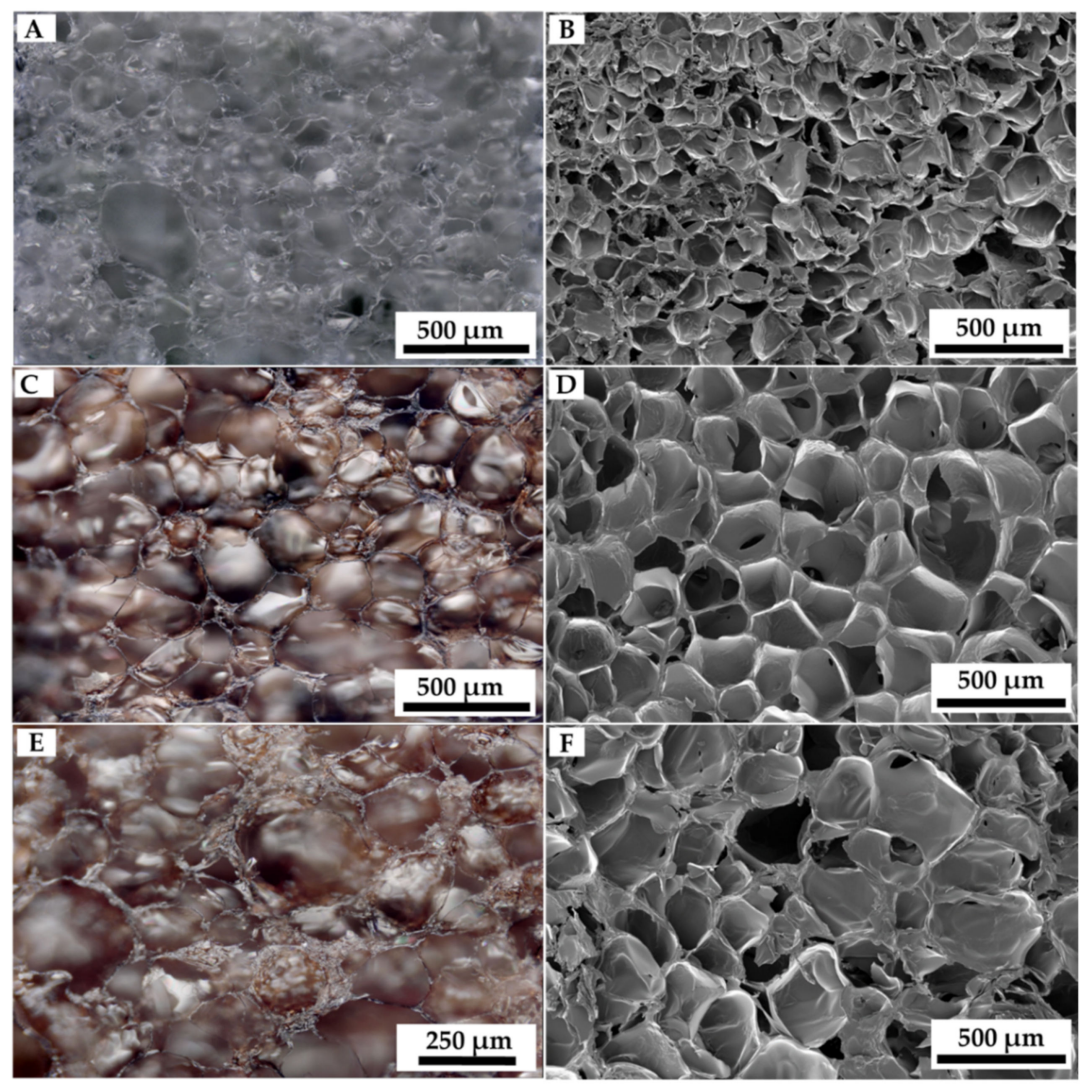
Optical microscopy (**left**) and SEM images (**right**) of some representative *G*_10_*C_z_N*_10_ gels as a function of the added catechol concentration. With (**A**,**B**) *G*_10_*N*_10_, (**C**,**D**) *G*_10_*C*_20_*N*_10_, (**E**,**F**) *G*_10_*C*_60_*N*_10_.

The preparation of the gels for morphological characterization will be described in the Section 4 below and is illustrated in Figure S6 of the Supporting Information. Note that the freeze-drying process was performed without any kind of additives. We also tried to freeze the gels and composite gels in liquid nitrogen, but this procedure induced intensive cracks in the gels, as displayed in Figure S7 of the Supplementary Materials. We found both by optical microscopy and by SEM that well-defined pores are formed for all the *G*_10_*C_z_N*_10_ and *G*_10_*D_z_N*_10_ investigated hydrogels. Some representative pictures are given in Figure 6 for the *G*_10_*C_z_N*_10_ hydrogels and in Figure S5 of the Supporting Information for the *G*_10_*D_z_N*_10_ hydrogels.

**Figure 7:**
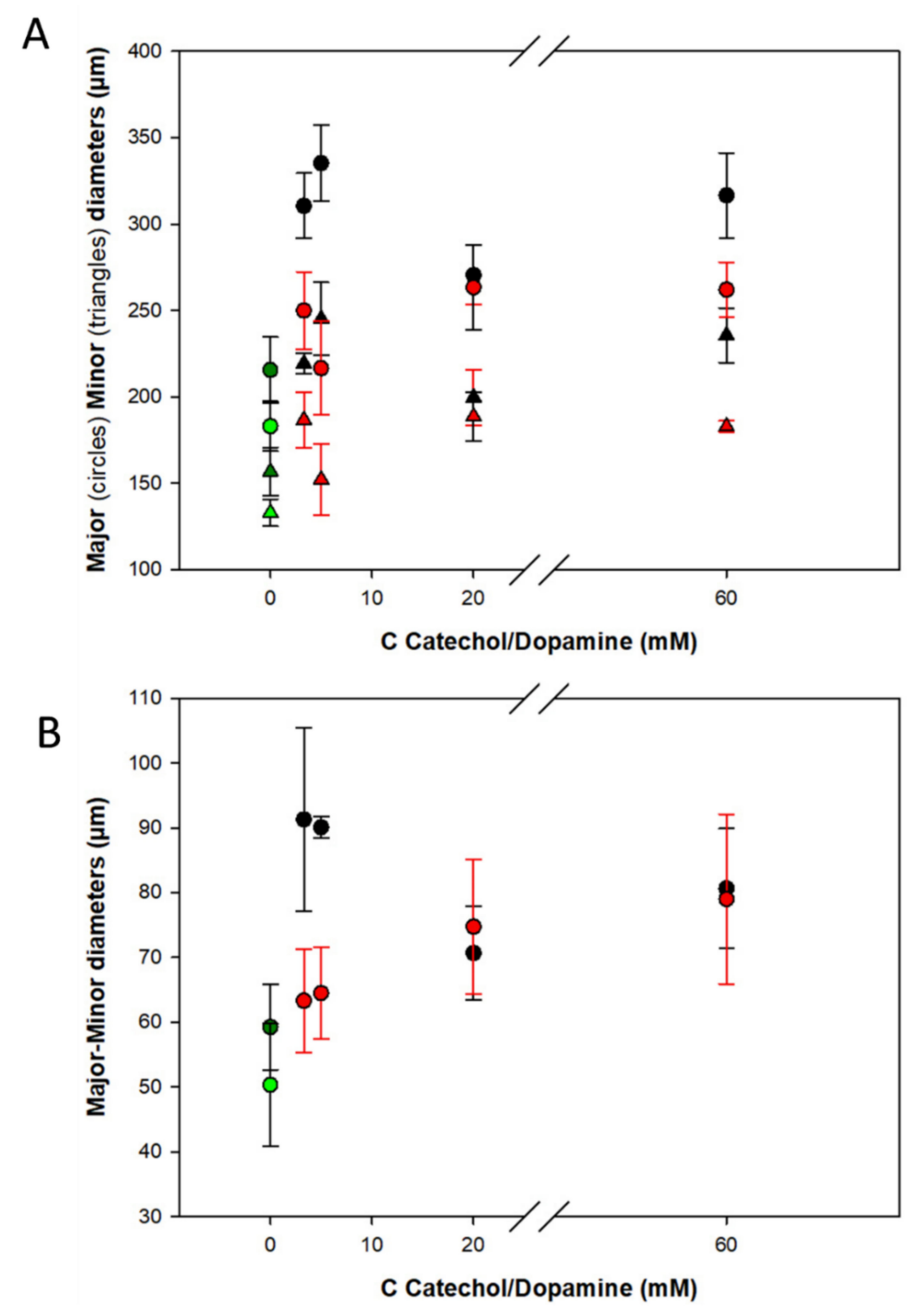
(**A**) Major (circles) and minor (triangles) pore diameters of freeze-dried samples as a function of phenolic compound concentration. (**B**) Difference between the major and the minor diameters taken from Figure 5A. *G*_10_ (dark green), *G*_10_*N*_10_ (green), and the catechol- (black) and dopamine-containing (red) hydrogels.

The largest pore size as well as the smallest pore size distribution were determined by analysis of the optical micrographs. It appears, for both kinds of gels, that the pore size increases slightly when the catechol or dopamine concentration increases from 0 to 5 mM. However, qualitatively (Figures S5 and S6 in the Supplementary Materials), the pore sizes seem almost unchanged for higher concentrations of added solutes. This qualitative trend is confirmed by a quantitative analysis of the pore size distribution made on the optical micrographs (Figure 7). The pores appeared to be non-spherical, as is apparent in both the optical and SEM micrographs (Figures S5 and S6 in the Supplementary Materials). Hence, the major and minor pore diameters as well as their differences, as estimators of an anisotropy factor, were measured by image analysis. It appears that within the experimental uncertainty, which was about 50 µm, both the major and minor pore diameters *reach a plateau value close to 250 and 175 µm, respectively, for a catechol or dopamine concentration close to 5 mM*. As a consequence, the anisotropy factor also plateaus at around 75 µm for the same catechol or dopamine concentration. A more detailed representation of the pore size distribution is given in Figure S8 of the Supplementary Materials, which confirms the trend given in Figure 7, namely an increase in the dimensions of the pores up to about 5 mM in added catechol or dopamine and a stabilization in the pore for higher concentrations. Additional information provided in Figure S8 of the Supplementary Materials is that the pore size distribution is very broad in the case of added catechol or dopamine; much broader than for the unmodified gels. Note that the gels made from both catechol or dopamine solutes behave similarly with respect to the pore diameter evolution. The catechol or dopamine concentration for which the pore sizes reach a plateau value, close to 5 mM, is lower than the concentration allowing for optimal elasticity, adhesion strength, and debonding energy, namely 20 mM (Figure 2). We hence strongly believe that the maxima found in both the gels’ elasticity and adhesiveness is due to an optimal catechol/NaIO_4_ or dopamine/NaIO_4_ ratio allowing for an optimal chemical post-oxidation reaction with nucleophiles present on the gelatin, and that the pore diameter is not an important parameter to explain the data shown in Figure 2 and Figure 3. Indeed, if a direct correlation would exist between the pore size and the gels elasticity and adhesiveness, one would have expected an initial decrease in these properties with the catechol or dopamine concentration before reaching a plateau [48]. This is obviously not the case (Figure 2).

As an additional remark: in none of the acquired images, or at the resolution of those images, did we observe solid particles which could originate from the oxidation process, in particular of dopamine. However, such very small particles or polymers, in the nanometer size range, may form in the presence of gelatin, as found in the presence of other, but non-gelling, proteins [47].

## 3. Conclusions

We showed that 10% *w*/*v* gelatin and 10 mM NaIO_4_ represent the ideal concentration of gelatin and oxidant for the purpose of studying the influence in phenolic compounds concentration on the gels’ mechanical and adhesive properties. The optimum was obtained at 20 mM of phenolic compounds of either catechol or dopamine. Below these values, it is likely that crosslinking has not been maximized and that the oxidizing environment has weakened the gelatin. Above these values, the loss in mechanical and adhesive properties may result from the disruption of the alpha helical contacts responsible for the gelation of gelatin due to the large number of phenolic compounds as well as the maintenance of an oxidizing environment. Our results show the adhesive strength of the *G*_10_*C*_20_*N*_10_ and of the *G*_10_*D*_20_*N*_10_ gels, specifically at 100 kPa, can compete with adhesives where the catechol moiety has been grafted to the polymer chains. Because their preparation is the result of a rapid (less than 5 min) one-pot process, the hydrogels investigated are ideal candidates for use as a biological glue. In the future, the swelling properties and the cytotoxicity of the catechol- or dopamine-containing hydrogels will be investigated, as well as their adhesiveness to tissues such as skin or muscles. In the present investigation, we focused on the adhesion on steel as a prototypal alloy, but the cytotoxicity aspect has not been considered yet. Indeed, even if the preparation of the adhesive gels is much easier than for those in which the catechol is grafted to the polymer, we may not exclude, particularly at high catechol or dopamine concentrations, that the gels release some none-covalently bound and cytotoxic species. As an additional perspective, the influence of condensed polyphenols such as resveratrol or epigallocatechin gallate on the mechanical and adhesive properties of the gelatin-based hydrogels is worth investigating.

## 4. Materials and Methods

### 4.1. Chemicals

All solutions were made from double distilled and deionized water (ρ = 18.2 MΩ cm, Millipore RO system). Pyrocatechol (ref. P0381), sodium periodate (ref. 311448), dopamine hydrochloride (ref. H8502), and gelatin (ref. G9382) were purchased from Sigma-Aldrich (Saint-Quentin-Fallavier, France). All products were used without further purification. The pH of the 50 mM sodium acetate (Merck chemicals) buffer was adjusted to 5.0 using concentrated hydrochloric acid. The mother solutions of pyrocatechol and dopamine were prepared during the heating process of the buffer solution, before the beginning of each gelation experiment.

### 4.2. Hydrogels Preparation

For the sake of simplicity, we will adopt the following nomenclature for the investigated hydrogels: GxCyNz or GxDyNz, where G, N, C, and D represent gelatin, sodium periodate (NaIO_4_), catechol, and dopamine, respectively. In this nomenclature, x, y, and z are the weight fraction of gelatin (% *w*/*v*), the molar concentration of catechol or dopamine, and the molar concentration of NaIO_4_, respectively. The volume of buffer solution has been adjusted (10, 9, or 8 mL) in order to reach a final and constant volume of 10 mL. The flask was closed and placed on a hot plate set at 75 °C. Gelatin powder was slowly added (to reach 10% or 20% *w*/*v*) and eventually a pyrocatechol or a dopamine solution was added. To start the oxidation process of catechol or dopamine, the concentrated NaIO_4_ solution was finally added. This concentrated NaIO_4_ solution was prepared at a 10-fold higher concentration than the expected final concentration. After homogenization with a magnetic stirrer (200 rpm), 0.6 mL of the hot mixture was used for rheological characterization in a cone plate geometry. However, 1.6 mL of the whole and hot sol was deposited on the cleaned stainless steel plate to evaluate the adhesion strength of the gels. The temperature of the horizontal plate was fixed at 25.0 ± 0.1 °C and controlled with a Peltier element. The deposition of the pre-gel mixture on the lower stainless steel plate corresponds to the beginning of the gelation process in all cases.

### 4.3. Characterization Methods

#### 4.3.1. Rheological Experiments

All rheological experiments were performed with a Kinexus Ultra rheometer (Malvern, Coventry, UK) fitted with a dynamometer limited to 50 N. The gelation kinetics and the temperature measurements were performed in the cone–plate geometry with an upper cone 4 cm in diameter and an angle of 174°. The distance between the apex of the cone and the lower plate was equal to 150 µm. However, the adhesion strength experiments were performed in a plate–plate geometry with an upper plate disk 2.0 cm in diameter. All the plates, cones, and disks were stainless steel. Before each experiment, they were cleaned with soap, hot distilled water, and ethanol. All the gelation kinetics were followed at a constant frequency of 1 Hz, at a constant strain of 1%, and at a constant temperature of 25 °C. The gelation kinetics were followed over the course of 3 h, and a new data point was performed every 30 s. After completion of some of the gelation kinetics, a temperature sweep experiment was performed from an initial temperature of 25 °C to a final temperature of 50 °C at a heating rate of 1.0 °C/min^−1^. The adhesion strength measurements were performed after 3 h of gelation, i.e., at the end of gelation kinetics, in the plate–plate geometry, the gap between the lower and the upper plate being constant and fixed at 1.0 mm. In these experiments, a compressive force of 1 N was applied during 1 s before retraction of the upper plate at a constant speed (100 µm/s). The force was measured every 10 ms up to separation, at which the force was equal to zero, corresponding to a complete rupture. A photograph of both plates was then taken in order to estimate if the rupture was of an adhesive (the gel being removed from one of the plates) or cohesive (gel parts being adherent on both plates) nature. The adhesive strength was obtained by dividing the maximal force obtained in such experiments by the area of the upper plate of the rheometer, specifically 3.14 cm^2^, neglecting its roughness. This yielded the adhesive strength of the corresponding gel. The specific energy of the rupture of the adhesive contact was calculated by integrating the area under the peeling curves after transformation of the time into a plate-to-plate distance and normalizing by the area of the upper plate, which was also 3.14 cm^2^.

#### 4.3.2. UV-Vis Spectra

UV-Vis spectra of freshly prepared solutions were measured with a double beam mc^2^ spectrophotometer (Safas, Monaco) in quartz cuvettes. The reference cuvette contained sodium acetate buffer (50 mM).

#### 4.3.3. FTIR-ATR Spectra

The spectra of samples were recorded using a Thermo Fisher Scientific Nicolet 380 (Breda, The Netherlands) FTIR spectrophotometer. The spectra of all samples were recorded in the wavenumber range 4000–400 cm^−1^, with a resolution equal to 4 cm^−1^. A total of thirty-two scans were performed for each of the samples. The results were processed using the OMNIC program (*Version 9.2.46*). The samples come from the powdering of hydrogels previously dried in the open air. The spectra were processed using Origin Pro 2022 software.

#### 4.3.4. Digital Microscopy

A VHX-5000 microscope (Keyence, Osaka, Japan) fitted with a VH-Z500R objective (Zoom Recognition Lens × 500 to 5000) and a white LED light source was used to image the same gels as those prepared for SEM characterization. Statistical analysis was performed with the Sigma-Plot software (Version 11.0). Pore size measurements were made on at least three different samples, with a minimum of 400 measurements per type of hydrogel.

#### 4.3.5. Scanning Electron Microscopy

First, we tried to freeze-dry the hydrogels after 5 min of freezing in liquid nitrogen, but this treatment induced extensive cracks in the obtained material, as shown in Figure S7 of the Supplementary Material. Hence we used another preparation method, as follows: 0.5 mL of pre-gels were poured into polypropylene cryotubes (Greiner Bio-One GmbH, Frickenhausen, Germany) ref. 122278 for −80 °C freezing. The gelation time at room temperature (closed containers) was adjusted to 24 h (based on the gelation rate of the weakest gels). Cryotubes were transferred in freeze-drying bottles provided with polypropylene tubes with round bottom to allow a more regular freezing and let frozen directly at −80 °C for a total of 4 h. Then, the samples were freeze-dried using Alpha 1-4LD plus (Christ, OsAterode, Germany) at 0.024 mbar (−54 °C in cold trap) for 16 h. This procedure is illustrated in Figure S6A of the Supporting Information. The obtained sponge samples were sectioned with a razor blade and mounted on stubs with carbon cement (Oxford Instruments, Gometz la Ville, France). Finally, they were coated with a HUMMER JR sputtering device (Technics, Union City, CA, USA) provided with gold palladium target. Scanning electron microscopy was performed with a FEI Quanta FEG 250 microscope (FEI Company, Eindhoven, The Netherlands) with an accelerating voltage of 5 kV.

## Figures and Tables

**Table 1:** Literature survey of the adhesion tests performed on catechol-containing hydrogels.

Adhesive Material	Substrates	Adhesion Strength	Ref.
**Dopamine-modified silk**	Aluminum	135 ± 15 kPa	[20]
**Mixture of proteins from Mytilus edulis**	Porcine skin	Dry conditions (25 °C, 40% relative humidity) after 24 h: 0.33 ± 0.17 MPa Wet conditions (37 °C, 80% relative humidity) after 24 h: 0.93 ± 0.32 MPa	[17]
**Complex coacervates made from mixtures of copolymers, the anionic one containing phosphate and dopamine groups, the cationic one containing primary amino groups.**	Cortical bone	120 kPa	[24]
**Poly((3,4-dihydroxymandelic acid)_x_-co-(lactic acid)_1−x_)**	Aluminum, dry state Aluminum, wet state Sanded steel, dry state Teflon, dry state	2.6 ± 0.4 Mpa 1.0 ± 0.3 MPa 1.7 ± 0.5 MPa 0.32 ± 0.05 MPa	[29]
**4-arm PEG (Mw = 10^4^ g·mol^−1^) modified with catechol + collagen (0.1% *w*/*v*) + hydroxyapatite (2.5% *w*/*v*). Crosslinking with 120 mM NaIO_4_.**	Skin tissue covered with blood	40 kPa	[28]
**4-arm PEG (150 mg·mL^−1^ + Laponite (0–2 wt%), NaIO_4_/dopamine ratio of 0.5**	Bovine pericardium	Laponite free gel: 3.5 ± 1.2 kPa	[25]
		Laponite at 2 wt%: 7.9 ± 1.8 kPa	
**4-arm PEG modified with dopamine or nitrodopamine**	Bovine pericardium, in wet conditions	Nitrodopamine: 4.0 ± 1.0 kPa at pH = 6.7 and 7.4 Dopamine: 1.8 ± 0.2 kPa at pH 6.7 8.0 ± 1.5 kPa at pH = 7.4	[26]
**4-arm PEG modified with dopamine, with NaIO_4_ loaded liposomes. Gelation was thermally triggered.**	Bovine skin	35 ± 12.5 kPa	[23]
**Chitin modified with pyrogallol, crosslinked with NaIO_4_**	Bovine skin	215 kPa	[27]
**Gelatin modified with 2,3,4-trihydroxybenzaldehyde**	PMMA	136.7 ± 1.4 kPa	[30]
	Iron	147.3 ± 6.3 kPa	
	Glass	92.9 ± 7.4 kPa	
	Pig skin	56.5 ± 4.4 kPa	
**Polymer containing dopamine and ureido-4-pirimidinoneside chains plus 2-ethyhexyl side chains (to induce phase microphase separation)**	Bovine pericardium	122.4 ± 42.4 kPa	[31]
**Alginate modified with 15% of the monomers modified with catechols.**	Titanium and gingiva	30 ± 5 kPa	[32]
**4-arm PEG modified with -NH_2_ or OH groups *mixed* with Tannic acid (TA)**	Epidermic side of porcine skin	TA+PEG-NH_2_: 0.17 ± 0.01 MPa TA-PEG-OH: 0.13 ± 0.01 MPa	[35]
**Poly(N-vinylpyrrolidone) *mixed* with Tannic acid**	Glass	3.71 MPa	[37]
**Gelatin *blended* with 20 mM pyrocatechol or 20 mM dopamine + 10 mM NaIO_4_, 3 h of gelation**	Stainless Steel	Dopamine: 89 ± 5 kPa Catechol: 99 ± 14 kPa	This investigation
**Gelatin *blended* with 10 mM pyrocatechol + 30 mM NaIO_4_, 3 h of gelation**	Stainless steel	80 ± 10 kPa	[38]

## Data Availability

The experimental data displayed in this article are available by contact with the main or the corresponding author.

## Supplementary Materials

The following supporting information can be downloaded at: https://www.mdpi.com/article/10.3390/gels8040210/s1, Figure S1: Gelation kinetics of gelatin gels at different gelatin concentrations. Figure S2: Final storage and loss moduli after 3 h of gelation monitoring for gelatin gels at variable concentrations from 6% to 30% + a constant concentration of NaIO4 (10 mM). Figure S3: Typical peeling curves for the catechol containing gels. Figure S4: Pictures of the gels at the end of the adhesive tests performed after 3 h of gelation. Figure S5: Optical microscopy and SEM images of some representative G10DYN10 gels as a function of the added dopamine concentration. Figure S6: Steps of freeze-drying of the gels before morphological characterization of their pores. Figure S7: Influence of liquid nitrogen freezing (5 min before freeze drying) on the shape of catechol modified gels. Figure S8: Size distribution of the major axes lengths (A and B) and of the minor axis lengths (C and D) for the G_10_ (dark green), G_10_N_10_ (green). Table S1: 1450 cm^−1^/1234 cm^−1^ peak intensity ratio in order to determine the disruption of α chains in the catechol/dopamine containing hydrogels. Table S2: Summary of the major, minor diameters and their difference for the different investigated hydrogels. Table S3: Composition of the different hydrogels investigated by means of electron microscopy.
