# Supplementary material for: Optimization of the Elasticity and Adhesion of Catechol- or Dopamine-Loaded Gelatin Gels under Oxidative Conditions
Source: Gels. 2022 Mar 31;8(4):210. doi: 10.3390/gels8040210 (PMC9028716; doi:10.3390/gels8040210)
Supplement: Supplementary file 1 [file gels-08-00210-s001.zip › gels-1632875-supplementary.pdf]

gels

Article

Optimization of the Elasticity and Adhesion of Catechol- or Dopamine-Loaded Gelatin Gels under Oxidative Conditions

Florence Back [1,2], Eric Mathieu [2], Cosette Betscha [2], Salima El Yakhlifi [1,2], Youri Arntz [1,2] and Vincent Ball [1,2,*]

1 Faculté de Chirurgie Dentaire, Université de Strasbourg, 8 Rue Sainte Elisabeth, 67000 Strasbourg, France; florence.back2@etu.unistra.fr (F.B.); salima.elyak@gmail.com (S.E.Y.); youri.arntz@unistra.fr (Y.A.)
2 Unité Mixte de Recherche 1121, Institut National de la Santé et de la Recherche Médicale, 1 Rue Eugène Boeckel, CEDEX, 67084 Strasbourg, France; ericmathieu74@yahoo.fr (E.M.); cosetta.betscha@medecine.u-strasbg.fr (C.B.)
* Correspondence: vball@unistra.fr

Supplementary Materials

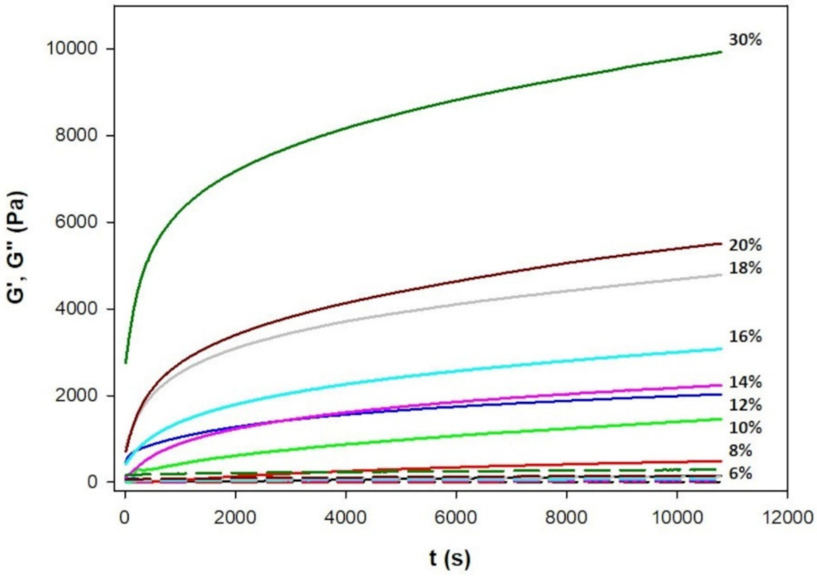

Figure S1. Gelation kinetics of gelatin gels from 6% to 30% w/v (from black to dark green lines as indicated) + $NaIO_4$ 10 mM. The storage moduli curves (G′) correspond to the full lines and the loss moduli curves (G″) correspond to the dashed lines.

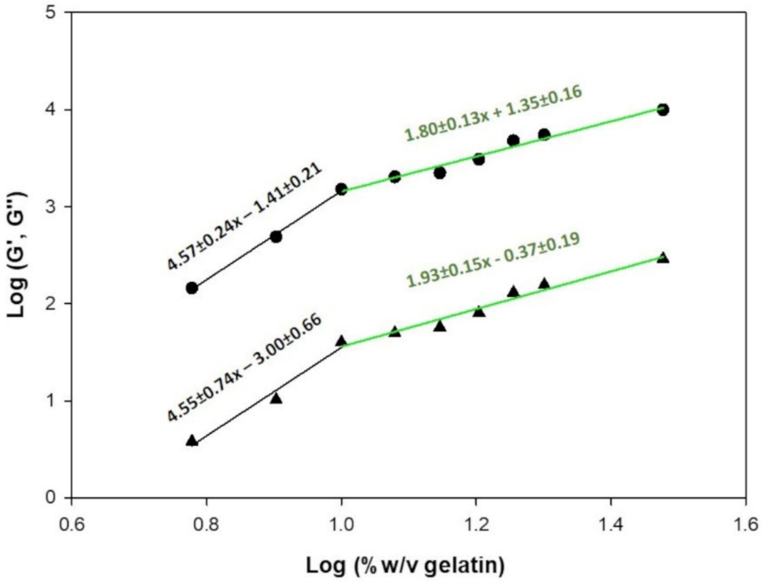

Figure S2. Final storage (black disks) and loss moduli (black triangles) after 3 h of gelation monitoring for gelatin gels at variable concentrations from 6% to 30% + a constant concentration of $NaIO_4$ (10 mM). The black lines represent the gelatin concentration from 6% to 10% and the green lines from 10% to 30%. The data above the line correspond to the slope (± one standard deviation) and the intercept (± one standard deviation) with the vertical axis respectively.

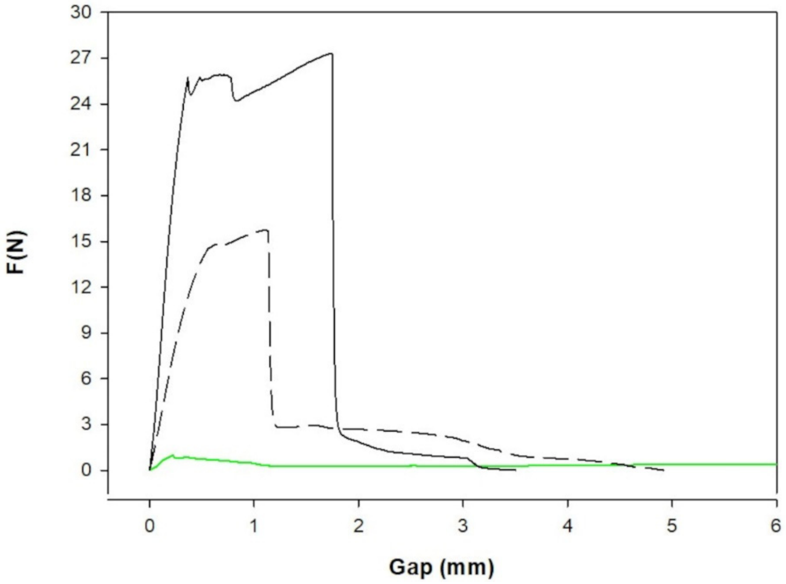

Figure S3. Typical peeling curves for $G_{10}N_{10}$ (green), $G_{10}C_{20}N_{10}$ (full line, black) and $G_{10}C_{60}N_{10}$ (dashed line, black) gels.

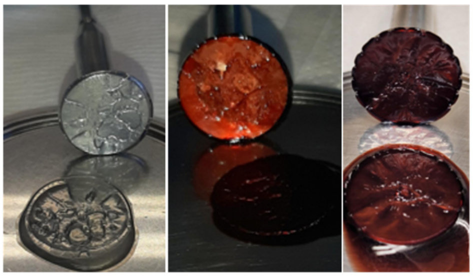

Figure S4. Pictures of the gels at the end of the adhesive tests performed after 3h of gelation. From the left to the right: $G_{10}N_{10}$, $G_{10}C_{20}N_{10}$ and $G_{10}C_{60}N_{10}$ gels.

Table S1. 1450 cm^{-1}/1234cm^{-1} peak intensity ratio in order to determine the disruption of α chains. With, right column (dopamine species ratios), left column (catechol species ratios) and middle columns (% differences from gelatin reference).

Peak intensity ratio (1450 cm^{-1}/1234 cm^{-1})					
		Difference (%)			
0.9484	G10N10	2.24	**0.00**	**G10 (ref.)**	**0.9701**
0.9699	G10C3.3N10	0.02	0.35	G10D3.3N10	0.9736
0.9932	G10C5N10	2.38	2.55	G10D5N10	0.9949
0.9815	G10C20N10	1.17	2.09	G10D20N10	0.9905
0.9972	G10C60N10	2.78	2.83	G10D60N10	0.9976
0.9821	G10C80N10	1.23	4.62	G10D80N10	1.0150

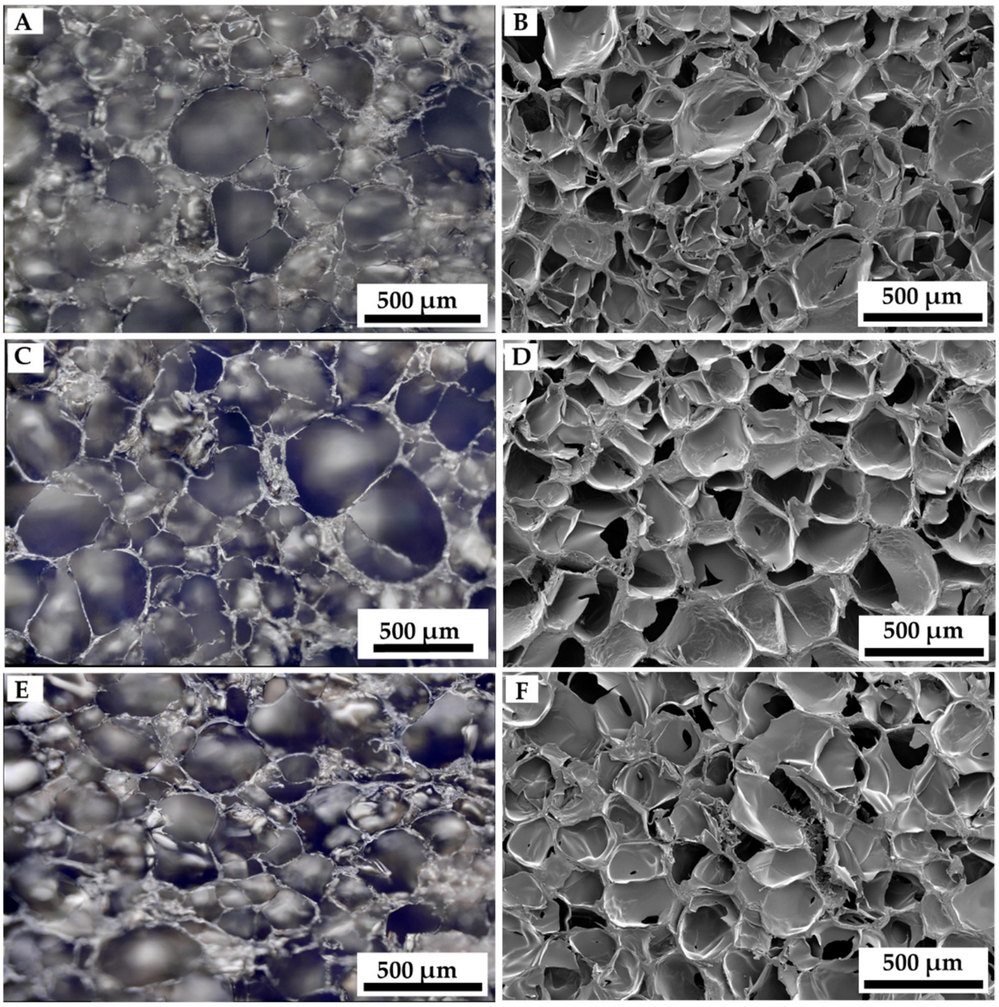

Figure S5. Optical microscopy and SEM images of some representative $G_{10}D_{Y}N_{10}$ gels as a function of the added dopamine concentration. With **A** and **B**, $G_{10}D_{3.3}N_{10}$, **C** and **D**, $G_{10}D_{20}N_{10}$, **E** and **F**, $G_{10}D_{60}N_{10}$.

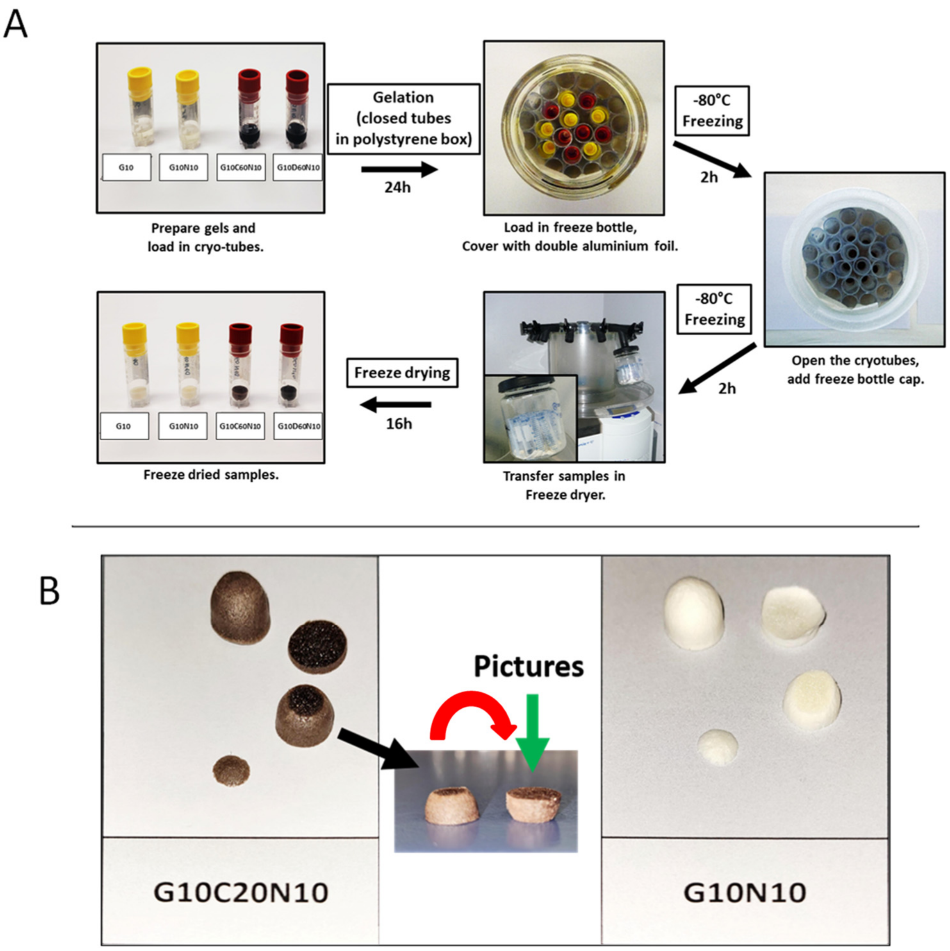

Figure S6. (**A**) Steps of freeze-drying of the gels before morphological characterization of their pores. (**B**) Examples of cut samples. View used for taking images (green arrow) and pictures of the $G_{10}C_{20}N_{10}$ and $G_{10}N_{10}$ samples.

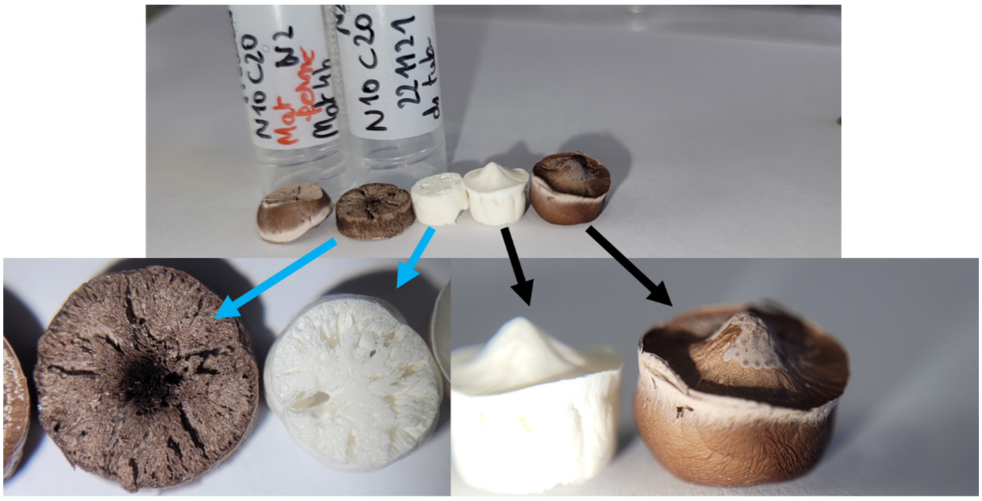

Figure S7. Influence of liquid nitrogen freezing (5 min before freeze drying) on the shape of catechol modified (brown) and unmodified gels (white). Many macroscopic cracks are appearing in the catechol modified gels.

Table S2. Summary of the major, minor diameters and their difference for the different investigated hydrogels. The measurements were made by optical microscopy as shown in Figure 6 and Figure 6 of the Supporting Material.

	Major (µm)	Minor (µm)	Major-Minor (µm)
**G10**	215±20	156 ±14	59±7
**G10N10**	183±15	133±8	50±10
**G10C3.3N10**	310±19	219±6	91±15
**G10D3.3N10**	250±22	186±16	63±8
**G10C5N10**	335±22	245±21	90±2
**G10D5N10**	217±28	152±21	64±8
**G10C20N10**	270±18	200±17	71±7
**GD20N10**	263±25	189±15	75±11
**G10C60N10**	317±25	236±16	81±10
**G10D60N10**	262±16	183±4	79±14

Table 3. Composition of the different hydrogels investigated by means of electron microscopy.

<table>
<tr><th colspan="5">Nomenclature</th></tr>
<tr><th rowspan="2">Name</th><th rowspan="2">% (w/v) Gelatin (G)</th><th colspan="2">(mM) Phenolic compounds</th><th rowspan="2">(mM) NaIO4 (N)</th></tr>
<tr><th>Dopamine hydrochloride (D)</th><th>Catechol (C)</th></tr>
<tr><td>G10</td><td rowspan="10">10</td><td></td><td></td><td></td></tr>
<tr><td>G10N10</td><td></td><td></td><td rowspan="9">10</td></tr>
<tr><td>G10C3.3N10</td><td></td><td>3.3</td></tr>
<tr><td>G10D3.3N10</td><td>3.3</td><td></td></tr>
<tr><td>G10C5N10</td><td></td><td>5</td></tr>
<tr><td>G10D5N10</td><td>5</td><td></td></tr>
<tr><td>G10C20N10</td><td></td><td>20</td></tr>
<tr><td>G10D20N10</td><td>20</td><td></td></tr>
<tr><td>G10C60N10</td><td></td><td>60</td></tr>
<tr><td>G10D60N10</td><td>60</td><td></td></tr>
</table>

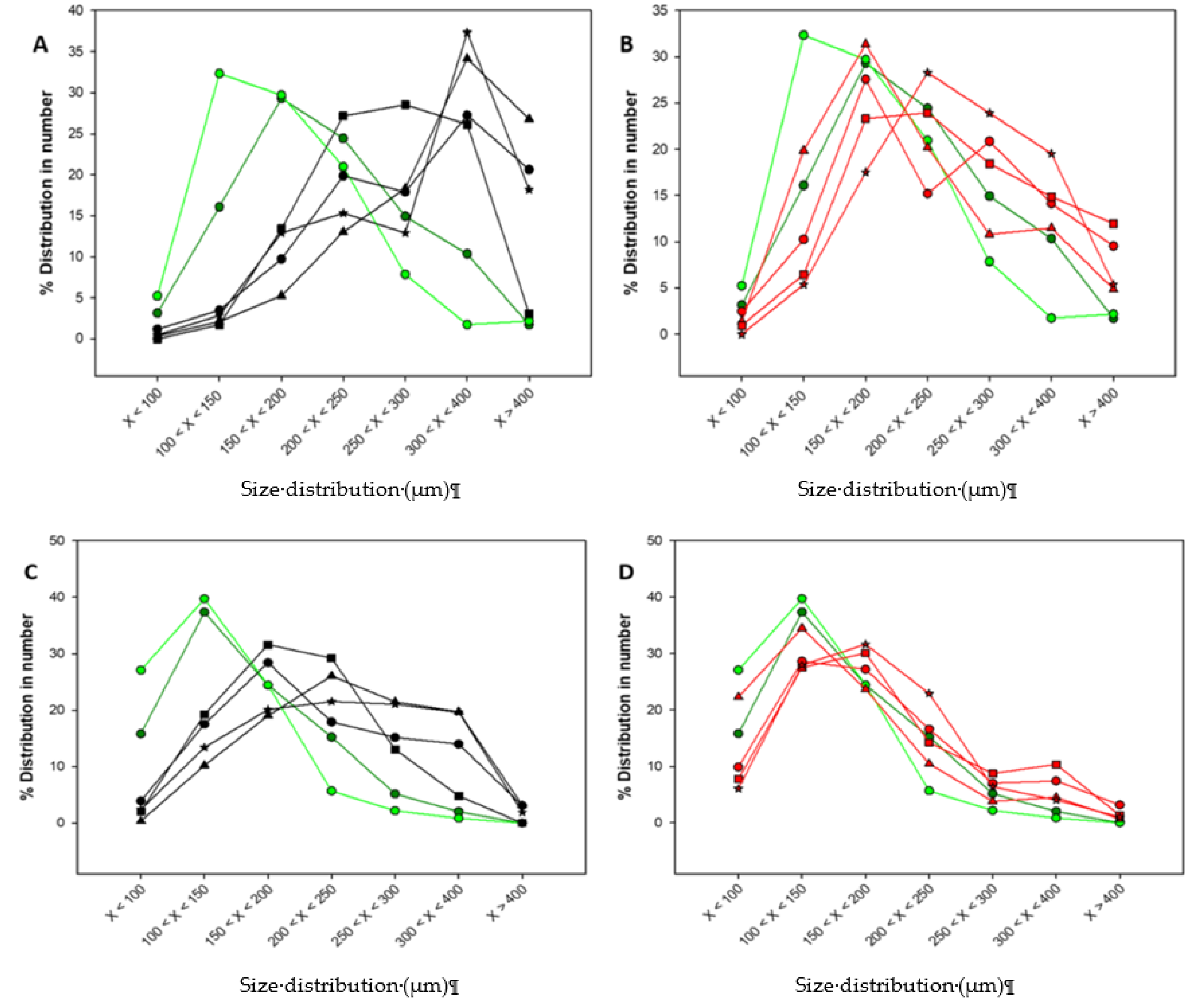

Figure S8. Size distribution of the major axes lengths (A and B) and of the minor axis lengths (C and D) for the G_{10} (dark green), $G_{10}N_{10}$ (green) and **A)** $G_{10}C_{3.3}N_{10}$ (black, circles), $G_{10}C_{5}N_{10}$ (black, triangles), $G_{10}C_{20}N_{10}$ (black, squares), $G_{10}C_{60}N_{10}$ (black, stars). **B)** $G_{10}D_{3.3}N_{10}$ (red, circles), $G_{10}D_{5}N_{10}$ (red, triangles), $G_{10}D_{20}N_{10}$ (red, squares) and $G_{10}D_{60}N_{10}$ (red, stars) hydrogels.
